# Prognostic Significance of COX-2 Immunohistochemical Expression in Colorectal Cancer: A Meta-Analysis of the Literature

**DOI:** 10.1371/journal.pone.0058891

**Published:** 2013-03-20

**Authors:** Ling Peng, Yun Zhou, Yina Wang, Haibo Mou, Qiong Zhao

**Affiliations:** 1 Department of Thoracic Oncology, The First Affiliated Hospital, School of Medicine, Zhejiang University, Hangzhou, China; 2 Zhejiang Food and Drug Administration, Hangzhou, China; Health Canada, Canada

## Abstract

**Background:**

Cyclooxygenase-2 (COX-2) is believed to be an important enzyme in the pathogenesis of colorectal cancer (CRC). Correlations between the expression of COX-2 with tumor growth and distant metastasis have become an issue; thus, attention has been paid to COX-2 as a prognostic factor. Various studies examined the relationship between COX-2 immunohistochemistry (IHC) overexpression with the clinical outcome in patients with colorectal cancer, but yielded conflicting results. The prognostic significance of COX-2 overexpression in colorectal cancer remains controversial.

**Methods:**

Electronic databases updated to October 2012 were searched to find relevant studies. A meta-analysis was conducted with eligible studies which quantitatively evaluated the relationship between COX-2 overexpression and survival of patients with colorectal cancer. Survival data were aggregated and quantitatively analyzed.

**Results:**

We performed a meta-analysis of 23 studies (n  =  4567 patients) that evaluated the correlation between COX-2 overexpression detected by IHC and survival in patients with colorectal cancer. Combined hazard ratios suggested that COX-2 overexpression had an unfavorable impact on overall survival (OS) (HR [hazard ratio]  =  1.193, 95% CI [confidence interval]: 1.02 ∼ 1.37), but not disease free survival (DFS) (HR  =  1.25, 95% CI: 0.99 ∼ 1.50) in patients with colorectal cancer.

**Conclusions:**

Cox-2 overexpression in colorectal cancer detected by IHC appears to have slightly worse overall survival. However, the prognostic value of COX-2 on survival in colorectal cancer still needs further large-scale prospective trials to be clarified.

## Introduction

Colorectal cancer is a leading cause of mortality in many countries [1]. The main prognostic factors in colorectal cancer are clinicopathological characteristics of the disease, including tumor size, stage, and grade. Although these parameters do reflect biological features of the tumor, they do not fully predict individual clinical outcome. There is the need for better markers to identify patients with poor prognosis. Researches have focused on the potential role of new biological factors involved in the carcinogenic process as prognostic markers to aid accurate prediction of clinical outcome of patients with colorectal cancer.

Much attention has been focused on the involvement of cyclooxygenase (COX) in tumor development and progression [2]. Cyclooxygenase with two known isoforms (COX-1 and COX-2) acts in the prostanoids biosynthesis pathway as a rate limiting enzyme [3]. COX-2 is an inducible enzyme that is upregulated in response to various stimuli, including cytokines, growth factor, and tumor promoters [4], [5]. Its pathophysiologic role has been associated with inflammation, wound healing, and carcinogenesis [6], [7]. It is known to be constitutively overexpressed and to have an oncogenic effect in a variety of cancers, including colorectal cancer [8], [9]. In colorectal cancer, cyclooxygenase-2 (COX-2) is overexpressed in the tumor tissue compared to the normal colonic mucosa [10].

Many retrospective studies have evaluated whether COX-2 overexpression may be a prognostic factor for survival in patients with colorectal cancer. However, the results of the studies are inconclusive and no consensus has been reached. It is necessary to establish whether COX-2 expression is a prognostic marker in colorectal cancer. In this meta-analysis, we collected and combined all eligible published articles about the relation between COX-2 and survival in colorectal cancer. The aim of our study was to test the hypothesis that COX-2 overexpression would predict the clinical outcomes of patients with colorectal cancer. We have used the statistical methods developed by Parmar *et al.* [11] to indirectly estimate hazard ratios and *P* values, enabling us to incorporate a number of studies in our meta-analysis. Moreover, we performed analysis of publication bias and heterogeneity between published studies.

## Materials and Methods

### Search Strategy and Study Selection

The electronic databases PubMed, EMBASE, Web of Science, and Cochrane Library were searched for studies to include in the meta-analysis. The upper date limit of Oct.31^st^, 2012 was applied, with no lower date limit. Searches include the terms “colorectal cancer” or “colon cancer” or “rectal cancer” or “colorectal carcinoma”, “COX-2” or “Cyclooxygenase-2”, and “prognosis”. The references cited by the included studies were also used to complete the search.

To be eligible for inclusion in this meta-analysis, a study must meet the following criteria: (1) investigate the association between COX-2 with patients’ prognosis (ie, disease free survival [DFS] and/or overall survival [OS]); (2) measure the expression of COX-2 with immunohistochemistry (IHC) in the primary colorectal cancer tissue (not in metastatic tissue or tissue adjacent to the tumor); (3) provide information on survival data; (4) have a median follow-up period no less than 24 months; (5) has been published as a full paper in the English language, and abstracts were excluded due to insufficient data to evaluate the methodological quality of the trial and/or to carry out meta-analysis; and (6) when the same author reported results from the same patient population, the most recent report or the most complete one was included. Abstracts of all candidate articles were read by two independent readers (LP and YZ). Articles that could not be categorized based on title and abstract alone were retrieved for full-text review. Disagreements were resolved by consensus between the two readers. During the process of thorough evaluation of the full articles we excluded all studies (1) with no more than 20 analyzed patients, (2) with insufficient data to calculate a hazard ratio (HR), (3) redundant multiple tested patient collectives. To determine the issue of multiple publications from the same data sets, we checked all author names, different institutions involved, and the time period of patient recruitment of the articles.

### Data Extraction

The final articles included were assessed independently by two readers (LP and YZ). Information was carefully retrieved from the full publications, using a standardized data collection form, including the following items: first author, year of publication, country of origin, number of patients analyzed, median age, gender distribution, cancer stage, preoperation treatment received, follow-up time, test method, cutoff value, antibody used, antibody working concentration, COX-2 positivity, and prognostic outcomes of interest (DFS and/or OS). If data from any of the above categories were not reported in the study, items were treated as “NS (not specified)”. Authors of the primary studies were not contacted for additional or unreported information. We did not use prespecified quality-related inclusion or exclusion criteria and did not weigh each study by a quality score, because the quality score has not received general agreement for use in a meta-analysis, especially observational studies.

### Statistical Methods

Included studies were divided into two groups for analysis: those with data regarding OS and those regarding DFS. For the quantitative aggregation of the survival results, we measured the impact of COX-2 overexpression on survival by HR between the two survival distributions. HRs and 95% confidence intervals (CIs) were used to combine as the effective value. If the HRs and their 95% CIs were given explicitly in the articles, we used crude ones. When these variables were not given explicitly, they were calculated from the available numerical data using methods reported by Parmar *et al.* [11].

Heterogeneity of the individual HRs was calculated with χ^2^ tests according to Peto’s method [12]. Heterogeneity test with inconsistency index (*Ι* ^2^) statistic and *Q* statistic was performed. If HRs were found to have fine homogeneity, a fixed effect model was used for secondary analysis; if not, a random-effect model was used. DerSimonian-Laird random effects analysis was used to estimate the effect of COX-2 overexpression on survival. Subgroup analyses were performed for survival endpoints (OS or DFS) and geographic settings.

Tissue studies reported data in a binary fashion, interpreting the COX-2 value as either “high” or “low”. The primary outcome for analysis was survival in patients with high COX-2 values as compared to those with low COX-2 values. By convention, an observed HR > 1 implies a worse prognosis in the high COX-2 expression group comparison to low COX-2 expression group. The impact of COX-2 on survival was considered to be statistically significant if the 95% CI did not overlap with 1. Horizontal lines represent 95% CI. Each box represents the HR point estimate, and its area is proportional to the weight of the study. The diamond (and broken line) represents the overall summary estimate, with CI represented by its width. The unbroken vertical line is at the null value (HR  =  1.0). For these analyses, a *P* value < 0.05 was considered to indicate significance.

Evidence of publication bias was sought of the pooled study groups using the methods of Egger *et al*. [13] and Begg *et al*. [14]. Intercept significance was determined by the *t* test suggested by Egger (*P* < 0.05 was considered representative of statistically significant publication bias). All of the calculations were performed by STATA version 11.0 (Stata Corporation, College Station, TX).

## Results

### Study Selection and Characteristics

**Figure 1:**
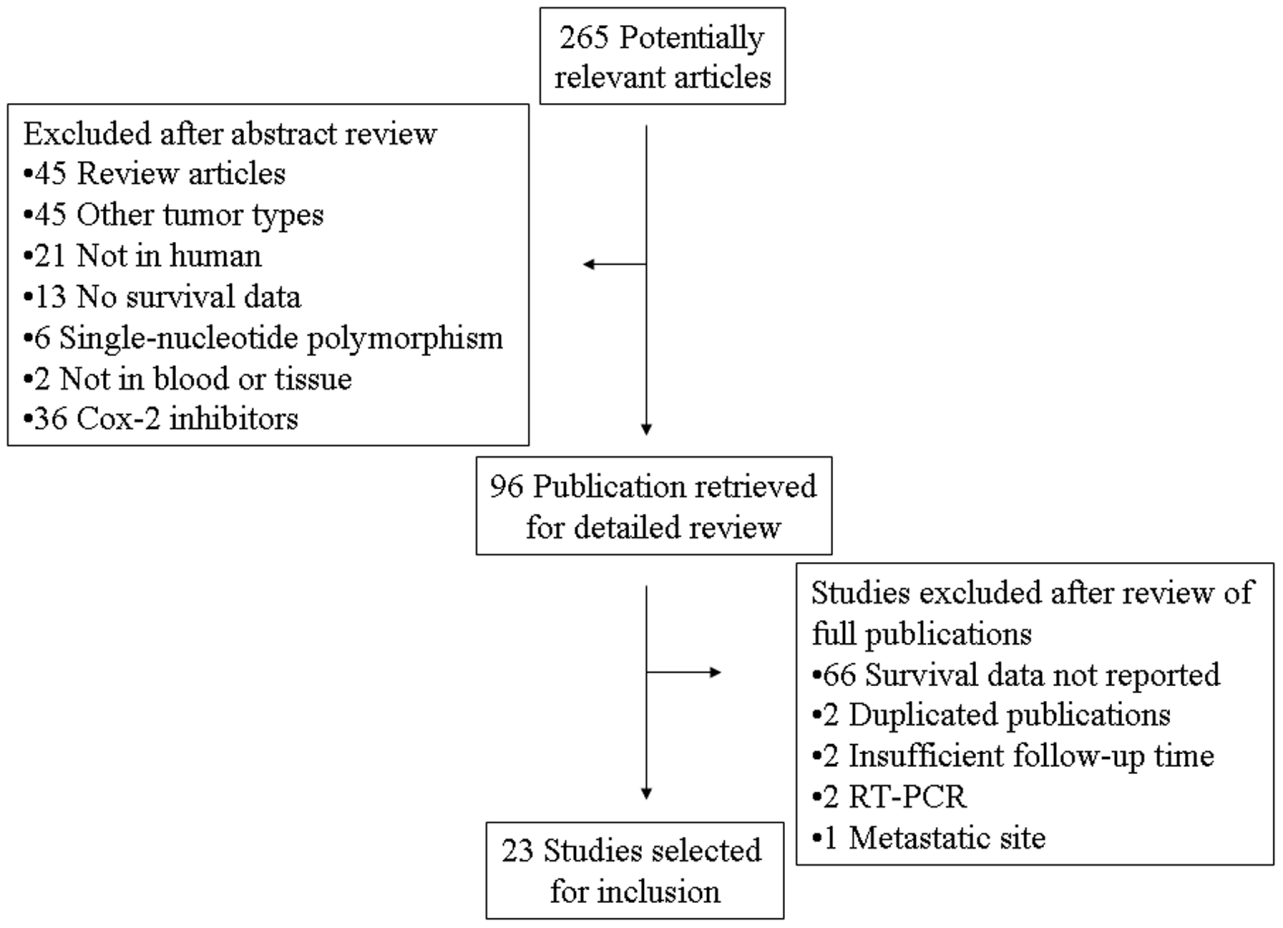
Flow chart of search strategy.

Two hundred and sixty five potentially relevant citations were reviewed, and 23 studies met inclusion criteria in the search strategy and study selection section, comprising 4567 patients for final analysis (Figure 1). The major baseline characteristics of the 23 eligible publications were reported in Table 1 and Table 2. The sample size of the included studies ranged from 35 to 747 patients (median sample size, 97.5 patients). The studies were conducted in 15 countries (China, Japan, Korea, France, Spain, Italy, Sweden, Germany, Netherlands, Turkey, Belgium, Saudi Arabia, Tunisia, Ireland, and the United States) and published between 1999 and 2012. Among the 23 studies, 13 studies (1591 patients, 34.8%) were performed in Asian populations, and the remaining 10 studies (2976 patients, 65.2%) followed non-Asian patients. All patients in the eligible studies were determined by pathological stage.

**Table 1:** Main Characteristics and Results of the Eligible Studies.

First Author	Year	Country of Origin	Recruitment Time	N	Male%	Age	Stage	Location	Preop treatment	Follow-up (Months)	COX-2 expression (%)
Miladi-Abdennadher [35]	2012	Tunisia	NS	35	NS	NS	I∼IV	Colorectal	NS	NS	65.7
Al-Maghrabi [36]	2012	Saudi Arabia	2005∼2009	94	51.1	59	I∼IV	Colon	NS	NS	56.4
Yoshinaga [31]	2011	Japan	2003∼2007	52	52	71.1	I∼IV	Colorectal	NS	NS	63.5
Pancione [37]	2009	Italy	1999∼2009	72	61.1	70.5	I∼IV	Colon	None	56	54.2
Inafuku [38]	2009	Japan	1996∼2005	109	55	NS	II	Colon	NS	NS	56
Debucquoy [39]	2009	Belgium	1996∼2003	99	64.6	63	NS	Rectal	RT±CT	42.4	80.8
Ogino [40]	2009	USA	1976∼2002	662	43	66.5	I∼IV	Colon	NS	NS	82.8
Lim [41]	2008	Korea	1992∼2001	231	48.5	61.2	I∼III	Colorectal	None	55.8	42.4
Chen [33]	2008	China	1999∼2001	96	60	54.8	I∼IV	Colorectal	None	NS	53.3
de Heer [25]	2007	Netherlands	1996∼1999	510	NS	NS	I∼III	Rectal	RT	NS	28
de Heer [25]	2007	Netherlands	1996∼1999	528	NS	NS	I∼III	Rectal	None	NS	33
Giralt [34]	2006	Spain	1994∼2001	74	66.7	64.8	Locally advanced	Rectal	RT±CT	53	51.4
Yamac [42]	2005	Turkey	1989∼1999	83	62.6	55	II∼IV	Colorectal	NS	37	62.6
Fux [43]	2005	Germany	1987∼1997	747	47.9	70.3	I∼IV	Colorectal	None	48.7	84.6
Zhan [44]	2004	China	1992∼2001	44	63.2	NS	I∼IV	Colorectal	NS	42	72.7
Soumaoro [45]	2004	Japan	1986∼1996	288	63.5	61	I∼IV	Colorectal	None	63	70.8
Wu [46]	2003	China	1993∼2001	139	50.4	59	I∼IV	Colorectal	NS	55	84.9
Buecher [47]	2003	France	1996∼1997	61	55.7	70.2	I∼II	Colorectal	NS	55.9	59
Zhang [48]	2002	Sweden	1972∼1996	112	NS	NS	I∼IV	Colorectal	NS	51.6	72.3
Yamauchi [49]	2002	Japan	1990∼1993	232	60.3	63	I∼III	Colorectal	NS	68	71.6
Joo [50]	2002	Korea	1995	60	58.3	60.7	I∼IV	Colorectal	None	72	70
Tomozawa [19]	2000	Japan	1990∼1994	63	60.3	NS	I∼III	Colorectal	NS	60	20.6
Masunaga [51]	2000	Japan	1990∼1999	100	59	NS	I∼IV	Colorectal	None	54	72
Sheehan [24]	1999	Ireland	1988∼1991	76	50	66.5	I∼IV	Colorectal	NS	32.4	81.6

**Table 2:** Main Characteristics and Results of the Eligible Studies (cont.d).

First Author	Method	Antibody	Working concentration	Cutoff	Outcome	Multivariate/Univariate	HR (95% CI)	Result
Miladi-Abdennadher [35]	IHC	Santa Cruz	1:100	CS	OS	Multivarivate	9.763 (1.629∼58.517)	Unfavorable
Al-Maghrabi [36]	IHC	Dako	1:50	CS	DFS	Surv curve	1.75 (0.69∼4.45)	Unfavorable
Yoshinaga [31]	IHC	IBL	1:500	1%	OS	Multivarivate	1.105 (0.066∼15.56)	Indeterminate
Pancione [37]	IHC	Cayman Chemical	1:200	50%	OS	Surv curve	1.64 (0.36∼7.38)	Indeterminate
Inafuku [38]	IHC	Dako	1:50	CS	DFS	Surv curve	0.99 (0.28∼3.44)	Indeterminate
Debucquoy [39]	IHC	Cayman Chemical	1:50	CS	OS	Surv curve	0.77 (0.03∼20.37)	Indeterminate
Ogino [40]	IHC	Cayman Chemical	1:300	CS	OS	Multivarivate	1.21 (0.87∼1.69)	Indeterminate
Lim [41]	IHC	Cayman Chemical	1:300	CS	OS	Multivarivate	1.096 (0.632∼1.899)	Indeterminate
Chen [33]	IHC	Beijing Golden Bridge	1:20	CS	OS	Surv curve	1.57 (0.35∼6.96)	Indeterminate
de Heer [25]	IHC	Cayman Chemical	1:100	CS	OS	Multivarivate	1.46 (1.10∼1.94)	Unfavorable
					DFS	Multivarivate	1.8 (1.2∼2.5)	Unfavorable
de Heer [25]	IHC	Cayman Chemical	1:100	CS	OS	Surv curve	1.10 (0.80∼1.51)	Indeterminate
					DFS	Surv curve	1.05 (0.79∼1.40)	Indeterminate
Giralt [34]	IHC	Novocastra	1:20	CS	DFS	Surv curve	1.88 (0.68∼5.25)	Indeterminate
Yamac [42]	IHC	Takara	1:500	CS	OS	Surv curve	0.99 (0.46∼2.14)	Indeterminate
					DFS	Surv curve	1.15 (0.40∼3.29)	Indeterminate
Fux [43]	IHC	Santa Cruz	1:50	CS	OS	Surv curve	1.30 (0.94∼1.81)	Indeterminate
Zhan [44]	IHC	NS	1:50	CS	OS	Multivarivate	2.248 (0.998∼5.114)	Indeterminate
Soumaoro [45]	IHC	Cayman Chemical	1:250	CS	OS	Multivarivate	4.114 (1.397∼12.120)	Unfavorable
Wu [46]	IHC	Cayman Chemical	1:200	CS	OS	Surv curve	0.76 (0.34∼1.67)	Indeterminate
Buecher [47]	IHC	Cayman Chemical	1:1000	5%	DFS	Multivarivate	2.13 (1.22∼3.73)	Unfavorable
Zhang [48]	IHC	Cayman Chemical	1:400	10%	OS	Surv curve	1.06 (0.41∼2.73)	Indeterminate
Yamauchi [49]	IHC	Alexis Corporation	1:1000	CS	DFS	Surv curve	2.05 (0.76∼5.53)	Unfavorable
Joo [50]	IHC	Cayman Chemical	1:500	CS	OS	Surv curve	1.05 (0.42∼2.64)	Indeterminate
Tomozawa [19]	IHC	IBL	1:40	CS	DFS	Multivarivate	10.086 (1.971∼51.612)	Unfavorable
Masunaga [51]	IHC	Cayman Chemical	1:300	CS	OS	Surv curve	0.98 (0.32∼2.96)	Indeterminate
Sheehan [24]	IHC	Cayman Chemical	1:500	1%	OS	Surv curve	0.86 (0.10∼7.33)	Indeterminate

Summary table of studies included in the meta-analysis. Results were either unfavorable (95% CI above 1.0) or indeterminate (95% CI crossing 1.0). Abbreviations: COX-2, Cyclooxygenase-2; N, number of patients; Surv curve: survival curve; IHC, immunohistochemistry; OS, overall survival; DFS, disease-free survival; CI, confidence interval; CS, complex score combining intensity and percentage; NS, not specified.

All of the studies reported the prognostic value of COX-2 status for survival in patients with colorectal cancer. Of the 23 studies, 9 directly reported HRs (multivariate analysis), while the other 14 studies provided survival curves. Among them, the proportion of patients exhibiting COX-2 overexpression in individual studies ranged from 20.6 to 84.9%. Estimation using survival curves were segregated according to either OS or DFS. A HR on OS and DFS could be extracted for 18 (17 publications) and 9 (8 publications) of studies, respectively.

Cyclooxygenase-2 positivity was associated with reduced OS or no statistically significant impact on OS in 3 and 15 studies, respectively. In the studies analyzing COX-2 overexpression on OS by multivariate analysis, 3 of the 7 studies suggested COX-2 overexpression indicated poor prognosis of OS, and 4 of the 7 studies resulted in an indeterminate role for COX-2 overexpression on OS. Among the 18 studies evaluating COX-2 overexpression of OS of CRC, 9 studies (1093 patients, 27.8%) were performed in Asian populations, and the remaining 9 studies (2841 patients, 72.2%) followed non-Asian patients. COX-2 overexpression was associated with reduced DFS or no statistically significant impact on DFS in 4 and 5 out of 9 studies. In subgroup analysis defined by geographic settings, 5 studies (581 patients, 33.1%) were performed in Asian populations, and 4 studies (1173 patients, 66.9%) followed non-Asian populations.

### Meta-Analysis

**Figure 2:**
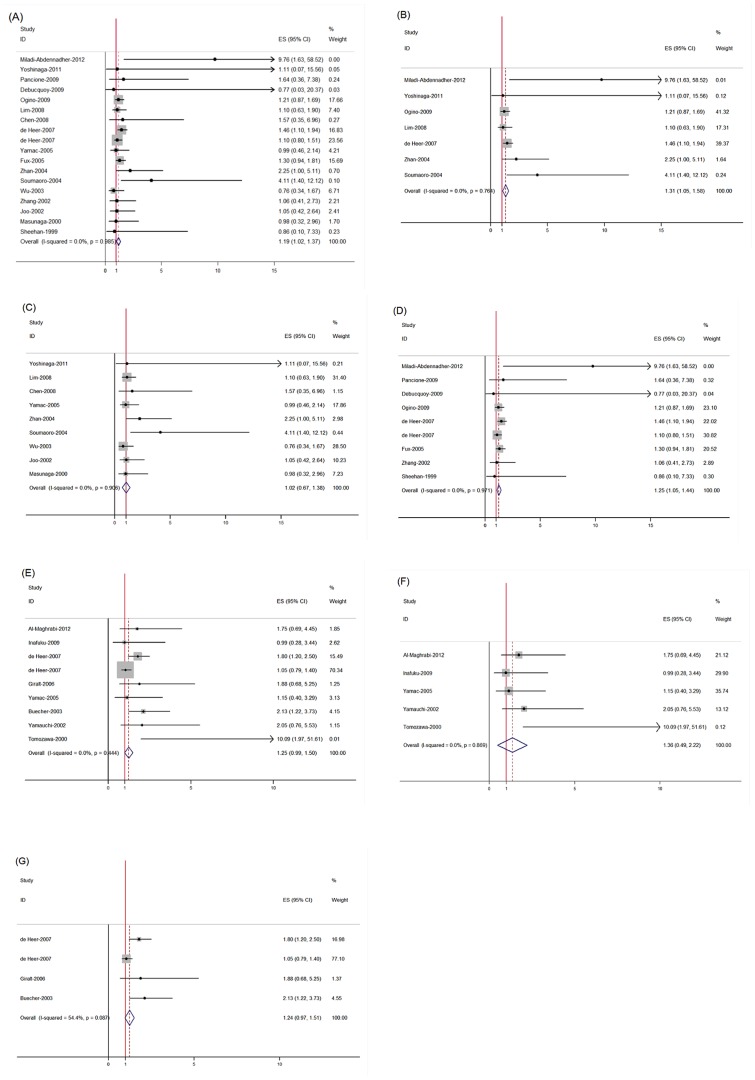
Meta-analysis (Forest plot) of the eligible studies evaluating the association between COX-2 overexpression and survival. Each study was shown by the name of the lead author and the HR with 95% CI. The summary HR and 95% CI were also shown (overall). (A) The 18 studies assessing COX-2 overexpression with OS in all population, (B) The 7 studies assessing COX-2 overexpression with OS with multivariate analysis, (C) The 9 studies assessing COX-2 overexpression with OS in Asian population, (D) The 9 studies assessing COX-2 overexpression with OS in non-Asian population, (E) The 9 studies assessing COX-2 overexpression with DFS in all population, (F) The 5 studies assessing COX-2 overexpression with DFS in Asian population. (G) The 4 studies assessing COX-2 overexpression with DFS in non-Asian population.

The results of the meta-analysis were shown in Table 3 and Figure 2. Overall, the combined HR for all 18 eligible studies (17 publications) evaluating COX-2 overexpression on OS was 1.19, (95% CI: 1.02 – 1.37), suggesting that COX-2 overexpression detected by IHC was an indicator of poor prognosis for colorectal cancer. No significant heterogeneity was observed among the studies. (*Q*  =  6.86, *I* ^2^  =  0%, *P*  =  0.985). The combined HR for 7 eligible studies evaluating COX-2 overexpression on OS by multivariate analysis was 1.31 (95% CI: 1.05 – 1.58), with no significant heterogeneity (*Q*  =  3.35, *I* ^2^  =  0%, *P*  =  0.764). When grouped according to geographic settings of individual studies, the combined HRs of Asian studies and non-Asian studies were 1.02 (95% CI: 0.67 – 1.38) and 1.25 (95% CI: 1.05 – 1.44), respectively, indicating COX-2 is an indicator of poor prognosis of OS in non-Asian patients but not in Asian patients. However, no statistically significant effect of COX-2 overexpression on DFS (HR  =  1.25, 95% CI: 0.99 – 1.50) in patients with colorectal cancer was observed. When grouped according to geographic settings, COX-2 overexpression had no significant impact on DFS both in Asian patients (HR  =  1.24, 95% CI: 0.97 – 1.51) and in non-Asian patients (HR  =  1.24, 95% CI: 0.97 – 1.51). No significant heterogeneity was observed among the studies on COX-2 overexpression on DFS (DFS in all patients [*Q*  =  7.90, *I* ^2^  =  0.0%, *P*  =  0.444], DFS in Asian population [*Q*  =  1.25, *I* ^2^  =  0.2%, *P*  =  0.869] and DFS in non-Asian population [*Q*  =  6.58, *I* ^2^  =  54.4%, *P*  =  0.087], respectively).

**Table 3:** Meta-analysis. HR value in subgroup analysis according to outcomes of interest, and population.

	N	Patients	HR (95% CI)	Heterogeneity Test (*Q*, *I^2^*, *P*)
COX-2 on OS	18	3934	1.19 (1.02 - 1.37)	6.86, 0.0%, 0.985
Multivariate analysis	7	1822	1.31 (1.05 – 1.58)	3.35, 0.0%, 0.764
Asian	9	1093	1.02 (0.67 – 1.38)	3.41, 0.0%, 0.906
Non-Asian	9	2841	1.25 (1.05 – 1.44)	2.28, 0.0%, 0.971
COX-2 on DFS	9	1754	1.25 (0.99 – 1.50)	7.90, 0.0%, 0.444
Asian	5	581	1.36 (0.49 – 2.22)	1.25, 0.0%, 0.869
Non-Asian	4	1173	1.24 (0.97 – 1.51)	6.58, 54.4%, 0.087

N: number of studies; HR: hazard ratio; CI: confidence interval.

### Publication Bias

**Figure 3:**
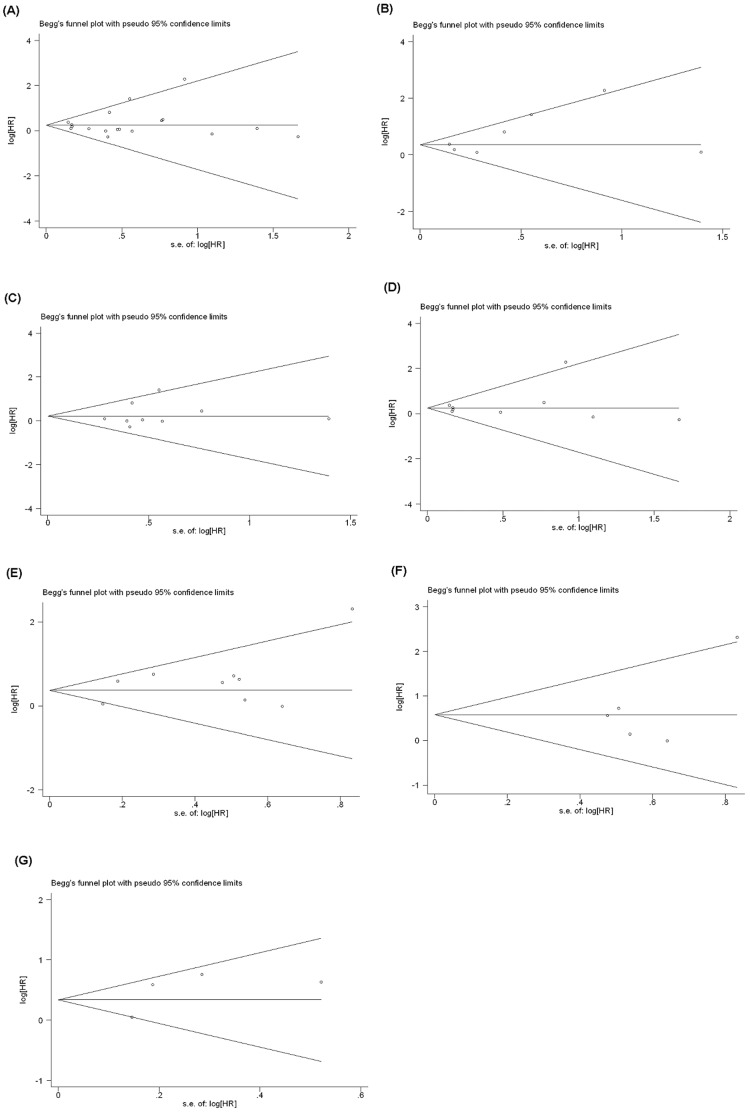
Funnel plot for studies included in the meta-analysis. Plots are arranged as follows: (A) COX-2 overexpression with OS, (B) COX-2 overexpression with OS by multivariate analysis, (C) COX-2 overexpression with OS in Asian population, (D) COX-2 overexpression with OS in non-Asian population, (E) COX-2 overexpression with DFS, (F) COX-2 overexpression with DFS in Asian population, and (G) COX-2 overexpression with DFS in non-Asian population.

Begg’s funnel plot and Egger’s test were performed to evaluate the publication bias of the eligible studies (Figure 3). Eighteen and nine studies investigating COX-2 overexpression on OS and DFS yielded an Egger’s test score of *P*  =  0.513 and *P*  =  0.153, respectively, indicating the absence of publication bias in the studies. Similar results were found for the subgroup analysis of COX-2 overexpression on OS in multivariate analysis (*P*  =  0.138), Asian (*P*  =  0.540) and non-Asian populations (*P*  =  0.553), and on DFS in Asian (*P*  =  0.303) and non-Asian populations (*P*  =  0.359), respectively. These results suggested that there were no publication biases in these subgroup analyses.

## Discussion

COX-2 has been shown to play an important role in carcinogenesis in various organ systems, such as colorectal cancer, breast cancer, lung cancer, esophageal cancer and pancreatic cancer [15]–[19]. The most extensive study of COX-2 was performed in colorectal cancer. COX-2 inhibitors (aspirin, nonsteroidal anti-inflammatory drugs, and celecoxib) have been shown to be effective in preventing colorectal cancer [20]–[22], which suggests a pathogenic role for COX in colonic tumorigenesis. Despite the well-accepted role of COX-2 in tumor development, studies are conflicting regarding prognostic significance of COX-2 in colorectal cancer. The presence of both significant and non-significant studies addressing the importance of COX-2 overexpression in colorectal cancer made it necessary to perform a quantitative aggregation of the survival results.

The present meta-analysis has combined 23 publications including 4567 patients to yield statistics, indicating a statistically significant role of COX-2 detected by IHC on overall survival in colorectal cancer, but not on disease-free survival. In subgroup analysis according to the different geographic settings of COX-2 on OS, results on OS were only significant with non-Asian populations. In our meta-analysis, patient cohorts were mainly from Eastern Asian countries (2976 patients, 65.2%). Although not entirely unexpected given the relatively high incidence of colorectal cancer in non-Asian populations, this raises the question whether the validity of results would also be applicable to Asian countries. The discrepant results of the primary studies are likely due to differences in patient cohorts, COX-2 detection methods, criteria for COX-2 expression, and multivariate survival analysis models. In our meta-analysis, the data were insufficient to determine the combined HR for subgroup divided according to disease stage, histology, or grade.

It has been reported that there were higher levels of COX-2 expression in patients with rectal cancer compared to patients whose tumors were located in the colon, possibly due to local variability in gene regulatory factors responsible for COX-2 expression [10]. Some other studies found no correlation between the location of colorectal tumor and COX-2 expression [23], [24]. However, in our meta-analysis, data were insufficient to analyze the association of location with COX-2 expression.

Our meta-analysis has several limitations. First, most of the included studies were retrospective studies, except that 2 studies are prospective studies [24], [25]. The level of evidence provided by retrospective studies was lower than that of randomized controlled trials. Secondly, prognostic markers are useful for identifying high-risk patients with poor prognosis, but a prognostic marker is preferably to be identified in the placebo arm of clinical trials. In our meta-analysis, the patient cohort were not randomized into treatment and placebo arms, therefore, the prognostic value of the biomarker should be interpreted with caution. Thirdly, although we did not detect significant heterogeneity among the primary studies, it is important to note that because of the small number of primary studies analyzed in each group, the power to detect potentially important differences is limited. Furthermore, the meta-analysis relied on publication, not on individual patient data (IPD); therefore, the multivariate analyses can’t be preformed. It is not known whether COX-2 expression is a prognostic factor, independently of other known prognostic factors, including tumor features and molecular characteristics, such as stage (tumor, node, metastasis), differentiation, age, sex and weight loss, and data on cancer treatment are limited in our meta-analysis. Therefore, the results must be interpreted with caution, because the IPD-based analysis provides the least bias and is more reliable than the literature-based meta-analysis [26].

COX-2 is proved to be an important role in the early stage of carcinogenesis in other cancer types, including colon cancer [27], [28]. There were several meta-analyses studying the prognostic value of COX-2 in other cancer types, such as lung cancer and esophageal cancer [29], [30]. The results suggest COX-2 could be of importance in early-stage NSCLC, and its impact might be lost at later steps because of the potential interaction with many factors [29]. In our meta-analysis, the data were insufficient to analyze COX-2 overexpression in early-stage colorectal cancer.

Association of COX-2 overexpression with poor outcomes provides a rationale for antitumor use in the treatment of colorectal cancer. COX-2 has become a therapeutic target for the treatment of colorectal cancer. In this meta-analysis, we have no detailed information about the use of NSAIDs (non-steroidal anti-inflammatory drug) of the patient cohorts. However, we know that NSAIDs only suppress COX-2 activity, but they have no effect on COX-2 expression. Therefore, we do not think that this factor may have any effect on the results. Moreover, the prognostic role of COX-2 in colorectal cancer should be examined in the context with other molecular markers. Some studies included in the meta-analysis had already addressed the association of COX-2 with other markers, such as peroxisome proliferator–activated receptors [31], p53 [32], β-catenin [33], VEGF [34].

Caution should be taken into account about biases. First, publication bias is a major concern in all forms of meta-analysis, as published studies are often positive [14]. As to the results of insignificant publication bias, we must point out that, when the sample size of the studies or the number of eligible studies is small, the power of detecting publication bias by linear regression model is reduced. Some important studies had to be excluded from our analysis, for reasons of small size, follow-up time, or insufficient survival data, etc. Although in the analysis obtained summary statistics did not support publication bias, language bias should not be completed avoided, because of restricted only in English. A selection process with rigid inclusion criteria was adopted in ascertaining studies, thereby reducing selection bias. Moreover, the method of survival data is a potential source of bias. If these statistics were not reported by the authors, we calculated from the data available in the article or by extrapolating them from the survival curves. These results should be confirmed by well designed prospective studies. However, few prospective prognostic studies concerning biomarkers have been reported. Another potential source of bias is the variable length of follow-up amongst studies. In order to overcome this potential bias, survival data in each study were extracted with the same observational period.

In conclusion, our meta-analysis is the first study to systematically estimate the association between COX-2 positivity and colorectal cancer survival. As determined in our meta-analysis, we concluded that COX-2 expression detected by IHC was associated with poor overall survival in colorectal cancer, but not disease-free survival. To strengthen our findings, well-designed prospective studies with better standardized assessment of prognostic markers should help to explore the relation between COX-2 overexpression and survival of colorectal cancer.
